# From Reads to Insights: Integrative Pipelines for Biological Interpretation of ATAC-seq Data

**DOI:** 10.1016/j.gpb.2021.06.002

**Published:** 2021-07-15

**Authors:** Ya Cui, Jason Sheng Li, Wei Li

**Affiliations:** Division of Computational Biomedicine, Department of Biological Chemistry, School of Medicine, University of California, Irvine, Irvine, CA 92697, USA

Underlying the regulation of mammalian gene expression at the level of transcription is the structure and modifications of chromatin. Understanding the twisting structures of DNA wrapped around histones and their higher-level ordering allows us to peek into a vast regulatory landscape. Looking closer, the physical accessibility of the genome provides information on the positions of nucleosomes and biologically-active regions — promoters, enhancers, insulators, and other regulatory elements. Currently, Assay for Transposase-Accessible Chromatin with high-throughput sequencing (ATAC-seq) is a widely used technology for detecting genome-wide chromatin accessibility [1], [2]. Compared to other methods, such as micrococcal nuclease sequencing (MNase-seq) [3], formaldehyde-assisted isolation of regulatory elements sequencing (FAIRE-seq) [4], and DNase I hypersensitive sites sequencing (DNase-seq) [5], ATAC-seq offers a simpler and quicker protocol by incorporating hyperactive Tn5 transposase to simultaneously cut open chromatin and ligate high-throughput sequencing adapters at chromatin-accessible regions [1], [2]. Importantly, ATAC-seq can also be applied to samples with limited starting cell material (500–50,000 cells) [1], [2].

These advantages of ATAC-seq — simplicity, speed, and low input material requirements — have made it highly popular in recent years. Publications and datasets using ATAC-seq have increased exponentially [6]. Several international consortia, including The Cancer Genome Atlas (TCGA) [7], CommonMind [8], FOUNDIN-PD [9], AD Knowledge Portal [10], and iPSCORE [11], use ATAC-seq data for population-scale studies. Despite the growing need to process ATAC-seq data, only a few analytical tools have been developed specifically for such data. Most of the current tools used for ATAC-seq data analysis are adopted from DNase-seq or chromatin immunoprecipitation sequencing (ChIP-seq) data analysis suites assuming similar data characteristics [12]. Typically, there are five steps for ATAC-seq data analysis: 1) quality control (QC); 2) read alignment; 3) peak calling; 4) downstream analysis (such as peak differential analysis, peak annotation, motif enrichment, and nucleosome position analysis); and 5) integration with multi-omics data [6]. However, there is no comprehensive and standalone analytical pipeline defined to guide ATAC-seq users.

In this issue, Liu et al. [13] and Qiu et al. [14] make great strides toward a standalone analytical ATAC-seq pipeline. Liu et al. present the ATAC-seq Integrative Analysis Package (AIAP), which defines a series of ATAC-seq-specific QC metrics to optimize data and further uses a pseudo single-end strategy (PE-asSE) specifically developed for ATAC-seq to improve data analysis [13]. Using these ATAC-seq-specific metrics (*e.g.*, reads under peak ratio, background, promoter enrichment, and subsampling enrichment) in conjunction with other traditional QC metrics, Liu et al. conducts QC checks at different steps of data processing and provide a user-friendly visualization of quality reports. Even further, Liu et al. incorporate a PE-asSE strategy to better analyze the Tn5 transposase insertion event within ATAC-seq data. Briefly, Liu et al. shift each end of the non-redundant uniquely mapped read pair +4 bp/−5 bp to define the Tn5 insertion position and then further extend 75 bp in both directions around the Tn5 insertion position. By applying this strategy, one non-redundant uniquely mapped read pair is divided into two single-end fragments. Applying the PE-asSE approach to ATAC-seq data of GM12878 cells leads to the identification of ∼ 99.9% peaks that can be identified by traditional strategies and an additional ∼ 23% (20,918) peaks that cannot be discovered by traditional methods. Further analysis shows that most of these PE-asSE-specific peaks overlap with known GM12878 DNase I hypersensitive sites (DHSs) and are highly enriched in all active histone modifications. Taken together, the additional open chromatin regions (OCRs) identified by PE-asSE are likely to be true functional regulatory elements rather than false positives, highlighting dramatic improvements in ATAC-seq OCR discovery. Given these improvements, Liu et al. further extend the usage of AIAP to differentially accessible region (DAR) analysis, applying AIAP to ATAC-seq data of mouse liver at embryonic day 11.5 (E11.5) and postnatal day 0 (P0). Peak differential analysis of data collected at these two time points results in an ∼ 35% increase in the number of DARs identified. Overall, Liu et al. present an ATAC-seq QC and analysis pipeline that dramatically improves the sensitivity of both peak calling and differential analysis.

In another paper of this issue, Qiu et al. present the Containerized Bioinformatics workflow for Reproducible ChIP/ATAC-seq Analysis (CoBRA), which provides a comprehensive and customizable ChIP and ATAC-seq analysis pipeline [14]. The strength of CoBRA is to integrate multiple commonly used functions, including normalization, copy number variation adjustment, sample clustering, differential peak calling, motif enrichment, Cistrome DB Toolkit [15] analysis, and Gene Set Enrichment Analysis (GSEA) [16] pathway analysis into the same package for scientists with limited computational experience. Based on the snakemake system [17], CoBRA can also easily integrate additional analytical tools in the future. CoBRA is well-documented and provides step-by-step tutorials for 3 case studies to guide users with limited computational experience. As an example, Qiu et al. have applied CoBRA to the ATAC-seq data of HL-60 promyelocytes differentiating into macrophages at five time points (0 h, 3 h, 24 h, 96 h, and 120 h). Unsupervised analysis results in three clusters showing clear differences in open chromatin between the early (0 h and 3 h), intermediate (24 h), and late stage (96 h and 120 h) time points. Further motif enrichment analysis in each cluster also identifies potential functional transcriptional regulators, such as early growth response protein (EGR) and Maf, in macrophage differentiation. Finally, Qiu et al. have integrated ATAC-seq data with sample-matched RNA-seq data, highlighting that genes differentially expressed during macrophage differentiation are flanked by changes in open chromatin structure.

In short, AIAP and CoBRA, the two useful ATAC-seq analysis pipelines developed by Liu et al. and Qiu et al., will provide a convenient entry and general guideline for scientists with limited computational experience to explore ATAC-seq data. Both pipelines use docker to allow compatibility on different operating systems and are able to generate high-quality figures automatically. These two studies represent significant progress in ATAC-seq analysis. Despite their benefits, more efforts are needed to extend the power of these ATAC-seq pipelines in the future. For example, more alternative tools may be included for each analytical step. Currently, both AIAP and CoBRA pipelines only include a popular software MACS2 [18] for peak calling, but not HMMRATAC [19], an alternative peak caller that is specifically developed for ATAC-seq and outperforms MACS2 [19]. Furthermore, to take advantage of multi-omics data from the same individual, more advanced analytical functions are required to integrate ATAC-seq data with other -omics data such as those from genotyping, RNA-seq, and ChIP-seq. For example, the integration of genotype and ATAC-seq modalities to identify quantitative trait loci for chromatin accessibility (caQTLs) has been recently used to evaluate genetic effects on chromatin accessibility [20]. caQTLs have been applied to the fine mapping of causal variants in noncoding chromatin accessible regions in multiple cell lines and tissues, such as blood [21], liver [22], and brain [23], furthering our understanding of regulatory mechanisms in human diseases at the tissue or cell type-specific levels. We expect more ATAC-seq-specific analytic tools to be developed in the future. Benchmarking studies for ATAC-seq analytical tools will help guide developers of comprehensive ATAC-seq pipelines in selecting the appropriate tools to be included. As ATAC-seq data continue to be generated for more individuals, cell types, and molecular processes, comprehensive and user-friendly ATAC-seq analytic pipelines will aid in the interpretation of biological mechanisms underlying ATAC-seq data.

## CRediT author statement

**Ya Cui:** Conceptualization, Writing – original draft, Writing - reviewing & editing, Supervision. **Jason Sheng Li:** Writing – original draft, Writing - reviewing & editing. **Wei Li:** Conceptualization, Writing - reviewing & editing, Funding acquisition, Supervision. All authors have read and approved the final manuscript.

2021 The Authors. Published by Elsevier B.V. and Science Press on behalf of Beijing Institute of Genomics, Chinese Academy of Sciences / China National Center for Bioinformation and Genetics Society of China.

This is an open access article under the CC BY license (http://creativecommons.org/licenses/by/4.0/).

Competing interests

The authors declare no competing financial interests.

### CRediT authorship contribution statement

**Ya Cui:** Conceptualization, Writing – original draft, Writing – review & editing, Supervision. **Jason Sheng Li:** Writing – original draft, Writing – review & editing. **Wei Li:** Conceptualization, Writing – review & editing, Funding acquisition, Supervision.
